# A three-dimensional analysis of fovea morphology in normal and diseased hips

**DOI:** 10.1093/jhps/hnaf046

**Published:** 2025-08-13

**Authors:** Vera M Stetzelberger, Jannine T Segessenmann, Cem Cek, Vlad Popa, Corinne A Zurmühle, Joseph M Schwab, Moritz Tannast

**Affiliations:** Department of Orthopaedic Surgery and Traumatology, HFR Fribourg Cantonal Hospital, University of Fribourg, Chemin des Pensionnats 2-6, 1708 Case Postale, Fribourg, Switzerland; Department of Orthopaedic Surgery and Traumatology, HFR Fribourg Cantonal Hospital, University of Fribourg, Chemin des Pensionnats 2-6, 1708 Case Postale, Fribourg, Switzerland; Department of Orthopaedic Surgery and Traumatology, HFR Fribourg Cantonal Hospital, University of Fribourg, Chemin des Pensionnats 2-6, 1708 Case Postale, Fribourg, Switzerland; Department of Orthopaedic Surgery and Traumatology, HFR Fribourg Cantonal Hospital, University of Fribourg, Chemin des Pensionnats 2-6, 1708 Case Postale, Fribourg, Switzerland; Department of Orthopaedic Surgery and Traumatology, HFR Fribourg Cantonal Hospital, University of Fribourg, Chemin des Pensionnats 2-6, 1708 Case Postale, Fribourg, Switzerland; Department of Orthopaedic Surgery and Traumatology, HFR Fribourg Cantonal Hospital, University of Fribourg, Chemin des Pensionnats 2-6, 1708 Case Postale, Fribourg, Switzerland; Department of Orthopaedic Surgery and Traumatology, HFR Fribourg Cantonal Hospital, University of Fribourg, Chemin des Pensionnats 2-6, 1708 Case Postale, Fribourg, Switzerland; Department of Orthopaedic Surgery and Traumatology, Inselspital Bern, University of Bern, Bern, Switzerland

## Abstract

The fovea capitis anchors the ligamentum teres on the femoral head. In normal hips, it resides within the acetabular fossa. However, clinical observations suggest that its position and morphology vary in pathological hips, potentially contributing to joint degeneration through fossa-foveolar mismatch (FFM). Understanding the fovea’s morphology is essential to clarifying its role in hip pathomechanics. We asked: (i) what is the proportion of the femoral head occupied by the fovea; (ii) what is the position of the fovea relative to the acetabular fossa; and (iii) what is the FFM index in a defined neutral position for different pathological and normal hips. Using three-dimensional models from computed tomography scans, we analysed 183 hips with femoroacetabular impingement or dysplasia and 22 with normal morphology. Using a standardized medial view of the fovea through the fossa, we determined: (i) the proportion of the fovea surface area on the femoral head; (ii) the positioning of the fovea relative to the fossa; and (iii) FFM indices in all study groups. (i) In normal hips, the fovea accounted for a median of 7% of the femoral head surface. (ii) While the fovea was positioned centrally in normal hips, dysplastic hips demonstrated an anterosuperior displacement of the fovea. (iii) Dysplastic hips had the highest FFM indices (median 0.13; *P* < .001). This study highlights variations in foveal morphology across different hip pathomorphologies. Foveal size was generally consistent. Foveal position differed markedly, with dysplastic hips showing anterosuperior displacement and the highest FFM indices. These results suggest that altered foveal morphology may contribute to pathological contact and degenerative lesions.

## Introduction

**Figure 1:**
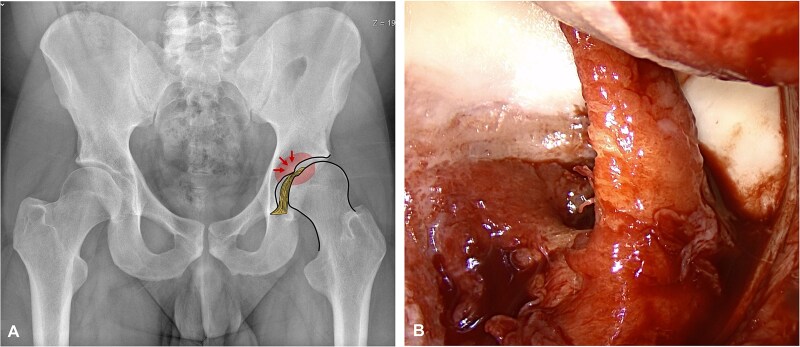
(A) The fovea capitis in this male patient with developmental dysplasia of the hip and coxa valga is located more superiorly and decentralized outside the acetabular fossa on the anteroposterior pelvic radiograph. (B) The intraoperative documentation from surgical hip dislocation shows a degenerated ligamentum teres and associated lesions on the acetabular fossa cartilage in this patient.

The fovea capitis, a cartilage-free area on the femoral head, serves as the attachment site for the ligamentum teres (LT) that is embedded within the acetabular fossa [1]. In our daily clinical practice, however, we sometimes observe that the fovea capitis is localized in a decentralized position outside the acetabular fossa (Fig. 1A). This displacement can lead to a pathological overlap of the fovea capitis onto the lunate surface in different motions or even in neutral position, and result in impingement of the LT (Fig. 1B). We recently introduced and validated a new biomechanical concept that quantifies this phenomenon: the fossa-foveolar mismatch (FFM) [2]. We hypothesize that the FFM may lead to early degeneration of the LT (Fig. 1B), the central acetabular cartilage, and the fovea capitis itself. Knowing the position of the fovea capitis is therefore important in evaluating young active patients with hip pain, because it could contribute to joint degeneration and play a role in correction osteotomies.

While the body of data is limited to descriptive anatomical and computed tomography (CT)-based studies [3–7], no study has evaluated the fovea’s position relative to the margins of the acetabular fossa. Additionally, the differences among acetabular and femoral morphologies have not been evaluated.

We therefore asked: (i) what is the proportion of the femoral head occupied by the fovea capitis; (ii) what is the position of the fovea relative to the acetabular fossa; and (iii) what is the FFM index in a defined neutral position for different hip pathomorphologies compared with a normal morphology group.

## Materials and methods

Our institutional review board (KEK Bern 2018-00078) approved the present study.

### Patients

**Figure 2:**
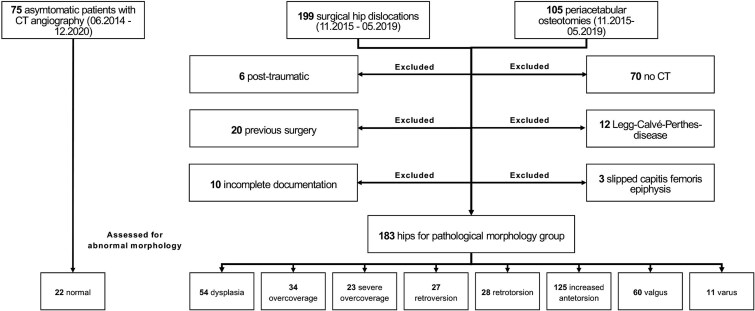
The flow diagram shows patient enrollment.

We reviewed 304 consecutive patients who underwent joint-preserving surgery for femoroacetabular impingement (FAI) (*n* = 199) or developmental dysplasia of the hip (DDH) (*n* = 105) at our institution between November 2015 and May 2019 (Fig. 2). Patients with posttraumatic hip pathomorphologies, slipped capital femoral epiphysis, Legg–Calvé–Perthes disease, incomplete documentation, or prior surgical interventions were excluded. This resulted in a final cohort of 183 hips with available preoperative CT scans from the pelvis to the femoral condyle. Standardized anteroposterior (AP) pelvic radiographs were obtained preoperatively for all patients, allowing allocation into study groups based on acetabular morphology and femoral torsion (Table 1). Overall, the mean age at the time of preoperative imaging was 28 ± 8 years old, with 20% being male (Table 2).

**Table 1:** Definition of the different study groups according to acetabular and femoral pathomorphology.

**Pathomorphology**		**Group**	**Definition**	***N* of hips (patients)**
Pathological	Overall			183 (169)
	Acetabular	Dysplasia	LCE angle < 22° and/or anterior coverage < 14% [8, 9]	54 (44)
		Overcoverage	LCE angle of 34°–39°, not all retroversion signs positive (posterior wall sign, cross-over sign, ischial spine sign) [8, 9]	34 (33)
		Severe overcoverage	LCE angle > 39 and/or femoral head touching/crossing the ilioischial line and/or total femoral coverage > 93% [8, 9]	23 (23)
		Retroversion	Crossover sign [10], ischial spine sign [11], and posterior wall sign [10] positive, retroversion index > 30%	27 (27)
	Femoral	Retrotorsion	Femoral torsion angle < 10° according to Murphy [12]	28 (28)
		Increased antetorsion	Femoral torsion angle > 25° according to Murphy [12]	125 (114)
		Valgus	Neck-shaft angle ≥140° [13]	60 (58)
		Varus	Neck-shaft angle ≤ 125° [13]	11 (11)
Normal		Normal group	Asymptomatic hips with CT scan for angiographic diagnostics and normal acetabular and femoral morphology: LCE angle 23°–33°, femoral torsion angle 10°–25° according to Murphy [12]	22 (22)

LCE: Lateral centre edge (°). Patients from the overall pathological group could be allocated to several acetabular or femoral pathomorphology subgroups.

**Table 2:** Demographic and clinical information of the study groups.

	**Pathological groups**								**Normal group**	
	**Acetabular morphology**				**Femoral morphology**					***P*-value**
**Variables**	**Dysplasia *n* = 54**	**Overcoverage *n* = 34**	**Severe overcoverage *n* = 23**	**Retroversion *n* = 27**	**Retrotorsion *n* = 28**	**Increased antetorsion *n* = 125**	**Valgus *n* = 60**	**Varus *n* = 11**	***n* = 22**	
***N* hips (patients)**	54 (44)	34 (33)	23 (23)	27 (27)	28 (24)	125 (114)	60 (58)	11 (10)	22 (22)	<.001
**Age at imaging (y)**	30 (18–50)	23 (16–54)	28 (18–50)	25 (16–36)	28 (15–54)	25 (16–54)	28 (15–65)	27 (18–54)	56 (37–66)	<.001
**Sex (% men)**	14 (26)	4 (21)	5 (20)	8 (30)	11 (39)	14 (11)	12 (20)	6 (55)	16 (73)	<.001
**Side (% right)**	35 (65)	12 (63)	11 (44)	15 (56)	16 (57)	76 (61)	40 (67)	9 (82)	12 (55)	.04
**BMI (kg/m** ^**2**^**)**	24 (18–39)	25 (19–36)	24 (17–34)	22 (17–34)	27 (20–36)	23 (17–39)	24 (18–34)	28 (21–33)	27 (19–33)	.001

Continuous variables are presented as median with range in parenthesis; categorical variables are presented as absolute numbers with percentage in parenthesis; Kruskal–Wallis test was used to compare the groups.

A ‘normal morphology’ group was defined using 22 asymptomatic individuals who had undergone pelvic CT scans for angiography at our institution between June 2014 and December 2020 (Fig. 2; Table 1). Inclusion for this group required normal acetabular and femoral morphology (Table 3).

**Table 3:** Radiological parameters of the study groups.

	**Pathological groups**								**Normal group**	
	**Acetabular morphology**				**Femoral morphology**					***P*-value**
**Variables**	**Dysplasia *n* = 54**	**Overcoverage *n* = 34**	**Severe overcoverage *n* = 23**	**Retroversion *n* = 27**	**Retrotorsion *n* = 28**	**Increased antetorsion *n* = 125**	**Valgus *n* = 60**	**Varus *n* = 11**	***n* = 22**	
**LCEA (°)**	15 (−17–22)	35 (34–38)	45 (39–61)	33 (9–51)	34 (19–55)	28 (−17–61)	28 (4–44)	37 (25–55)	34 (25–39)	<.001
**Extrusion index (%)**	33 (22–67)	15 (11–18)	8 (−4–17)	18 (2–41)	17 (−4–29)	22 (−4–67)	22 (8–46)	13 (−4–26)	18 (11–26)	<.001
**Acetabular index (%)**	14 (2–31)	2 (−11–11)	−6 (−14–5)	2 (−10–19)	4 (−11–13)	5 (−19–31)	14 (0–54)	1 (−11–9)	5 (−6–9)	<.001
**Crossover sign (% positive)**	37 (69)	17 (90)	21 (84)	27 (100)	23 (82)	99 (79)	47 (78)	9 (81)	17 (77)	.11
**PW sign** **(% positive)**	44 (82)	9 (47)	12 (48)	27 (100)	17 (61)	76 (61)	34 (57)	7 (64)	14 (64)	<.001
**Ischial spine sign (% positive)**	20 (37)	7 (37)	13 (52)	26 (96)	10 (37)	50 (40)	22 (34)	5 (46)	0 (0)	<.001
**Retroversion index (%)**	3 (0–54)	7 (0–39)	14 (0–54)	41 (31–77)	4 (0–77)	9 (0–57)	14 (0–54)	5 (0–43)	3 (0–70)	<.001
**CCD angle (°)**	137 (126–157)	137 (122–161)	135 (117–153)	136 (120–157)	135 (117–146)	137 (121–161)	144 (140–161)	124 (117–125)	134 (124–140)	.07
**Alpha angle (°)**	52 (34–120)	48 (36–75)	45 (31–73)	51 (30–79)	53 (31–75)	50 (30–120)	49 (34–120)	53 (31–74)	n.a.	.002
**Femoral torsion angle (°)**	39 (−5–68)	35 (−11–60)	31 (1–56)	33 (0–68)	2 (−11–8)	41 (26–68)	40 (0–63)	6 (−8–31)	20 (10–25)	<.001

Continuous variables are presented as median with range in parenthesis; categorical variables are presented as absolute numbers with percentage in parenthesis; Kruskal–Wallis test was used to compare the groups.

### Three-dimensional model

We generated a three-dimensional (3D) surface model of the pelvis and femur using the semiautomatic segmentation software Amira (Thermo Fisher Scientific 2019.3, Waltham, MA, USA). We inspected the inner side of the acetabulum from all angles after removing the femoral head from the 3D model. The acetabular fossa borders were defined based on the visible depression and contour; subsequently, using the segmentation editor, we removed the complete acetabular fossa.

**Figure 3:**
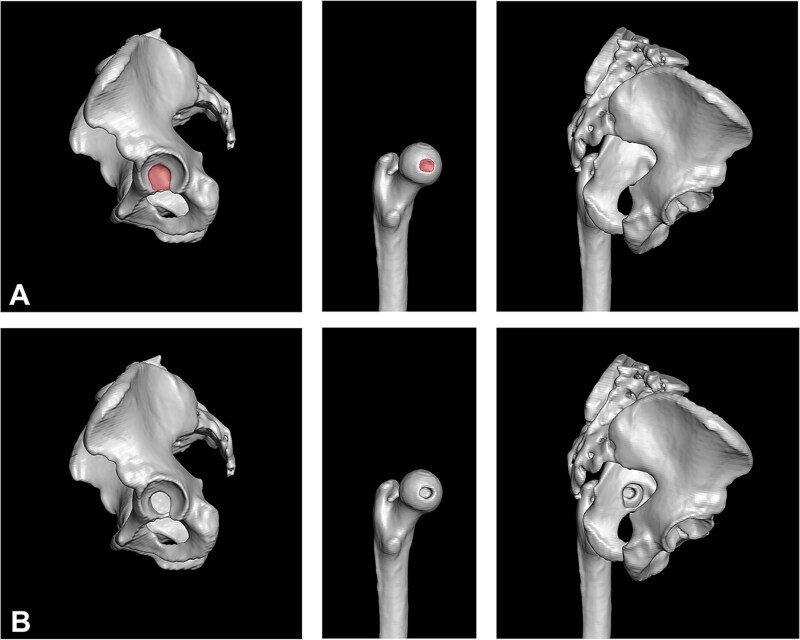
(A and B) The three-dimensional model is built based on the CT scans of the pelvis and femur. (A) The acetabular fossa and fovea capitis are identified. (B) The fossa is excised and the fovea is manually deepened.

Similarly, we identified the borders of the fovea capitis precisely, through detailed visual inspection of the 3D-reconstructed femoral head surface in the Amira software (Fig. 3A and B). The model was rotated and assessed from multiple angles to accurately delineate the anatomical rim of the fovea based on its surface concavity and surrounding morphology. For better contrast with the projected border of the acetabular fossa, the depression of the fovea was artificially increased. To achieve this, we used Amira’s ‘Create Surface Geometry’ tool to introduce a cylindrical defect (diameter approximating the best-fitting circle of the fovea border, depth ~ 10 mm). This was done by manually positioning a virtual cylinder over the foveal region and subtracting this volume from the femoral head surface model—effectively mimicking a deeper foveal depression.

**Figure 4:**
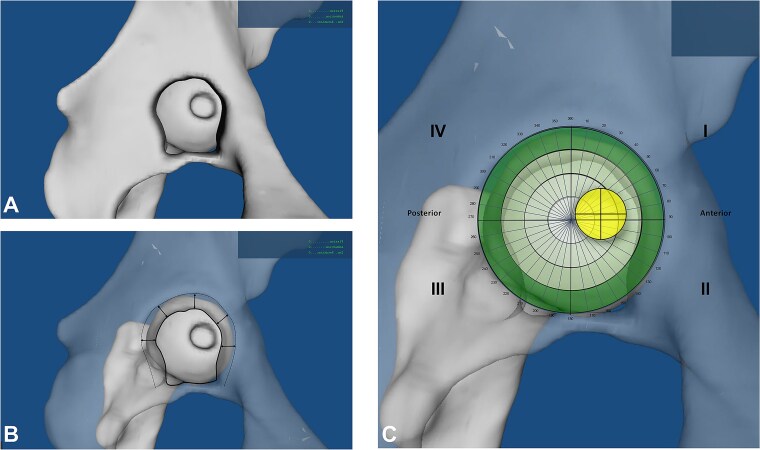
(A and B) The pelvis is made transparent and the 3D model is positioned in a standardized way (ligamentum transversum in the exact horizontal plane; the fovea equidistant from the acetabular diameter). (C) The position of the fovea is determined using quadrants and radial zones.

**Figure 5:**
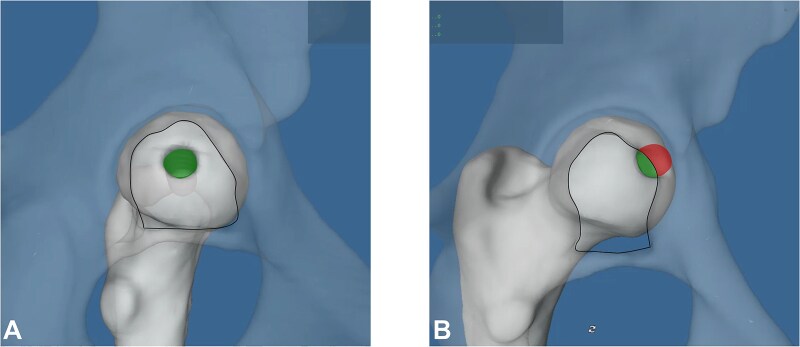
(A and B) The fossa-foveolar mismatch (FFM) index is calculated for each hip by dividing the surface area located outside the acetabular fossa by the total surface area. (A) This specific hip has a FFM index of 0. (B) This patient with an increased femoral antetorsion exhibits a high FFM index.

Then the pelvis was made 50% transparent for better visualization of the fovea in case it was located outside the acetabular fossa (Fig. 4A and B). A direct medial view of the femoral head through the excised acetabular fossa was used for all measurements (Fig. 4A–C). The pelvis was oriented such that the origin of the transverse ligament at the acetabular fossa was aligned horizontally, and the femoral head was positioned centrally within the acetabulum. The femoral condyles were automatically oriented in the frontal plane in all hips. (1) The surface area of the fovea, the femoral head, and the acetabular fossa were digitally determined. Using the femoral head diameter on the CT scans, we calculated the exact surface area of the femoral head, which allowed determination of the exact surface area of the fovea and the proportion taken by the fovea on the femoral head. (2) We drew a circle corresponding to the diameter of the femoral head and measured in which position the centre of the fovea was located using a 360° coordinate system, which allowed allocation of each fovea to a quadrant (Fig. 4A–C). The circle was furthermore divided in four radial zones to determine how central the fovea was positioned relative to the centre of the acetabular fossa. The third and fourth radial zones were combined in the final analysis. (3) The FFM index was calculated using the previously outlined method [2], by dividing the surface area of the fovea located outside the fossa by its total surface area in the defined neutral position (Fig. 5A and B).

### Statistical analysis

Normal distribution for continuous demographic, clinical, radiological variables, and the outcome variables was tested using the Kolmogorov–Smirnov test. (1) The proportion of the fovea on the femoral head was compared among the nine groups using the Kruskal–Wallis test. Each pathological study group was compared with the normal group using the Wilcoxon test. (2) The fovea position (defined by a specific quadrant and corresponding radial zone) of each hip was compared using the chi-squared test. Each pathological morphology study group was compared with the normal morphology group using the same test. (3) FFM indices for the fovea’s neutral position were compared using the Kruskal–Wallis test. Each pathological morphology study group was compared with the normal morphology group using the Wilcoxon test.

To control for multiple comparisons, we performed a Bonferroni correction, resulting in an adjusted significance level of α = 0.006 (0.05/8 with 8 being the number of comparisons). Results are presented as medians with range in parentheses and a *P*-value < .006 was considered statistically significant.

## Results

**Figure 6:**
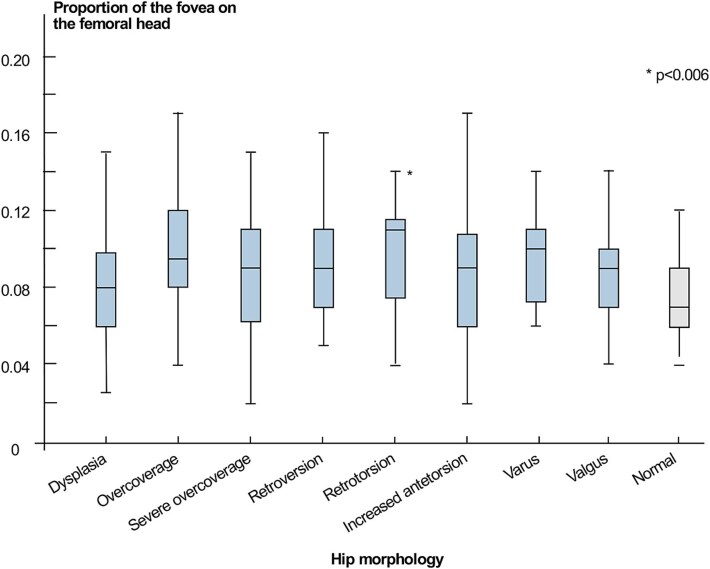
The boxplot shows the proportion of the fovea surface area on the femoral head for all groups. *: Significantly different compared with the normal group.

(1) In the normal morphology group, the surface area of the fovea accounted for a median 7% (4%–15%) of the surface area of the femoral head. By comparison, the retrotorsion group exhibited a median 11% (4%–15%; *P* = .003; Table 4; Fig. 6).

**Table 4:** Results of the morphological analysis of the fovea.

	**Pathological groups**								**Normal group**	**P-value**
	**Acetabular morphology**				**Femoral morphology**					
**Variables**	**Dysplasia *n* = 54**	**Overcoverage *n* = 34**	**Severe overcoverage *n* = 23**	**Retroversion *n* = 27**	**Retrotorsion *n* = 28**	**Increased antetorsion *n* = 125**	**Valgus *n* = 60**	**Varus *n* = 11**	***n* = 22**	
**Proportion of the fovea on the femoral head**	0.08 (0.02–0.15)	0.095 (0.04–0.17)	0.09 (0.02–0.15)	0.09 (0.05–0.16)	0.11 (0.04–0.15)*	0.09 (0.02–0.17)	0.09 (0.04–0.14)	0.1 (0.06–0.14)	0.076 (0.04–0.15)	.03
**Foveal radial zones**										
**1 (central)**	14 (26)*	5 (26)*	13 (52)*	18 (67)	24 (86)	22 (18)*	16 (27)*	11 (100)	22 (100)	<.001
**2 (intermediate)**	27 (50)*	13 (68)*	12 (48)*	5 (19)	3 (11)	76 (21)	35 (58)*	0	0	<.001
**3 or 4 (peripheral)**	13 (24)	1 (5)	0	4 (15)	1 (4)	21 (22)	9 (15)	0	0	<.001
**Foveal quadrant**										
**Anterosuperior (I) *n* (%)**	36 (65)*	13 (38)	10 (43)*	12 (40)	8 (29)	59 (48)*	33 (55)*	1 (9)	2 (9)	<.001
**Anteroinferior (II) *n* (%)**	14 (25)*	16 (47)	13 (57)	11 (37)	4 (14)*	59 (48)	23 (38)	3 (27)*	13 (59)	<.001
**Posteroinferior (III) *n* (%)**	0	2 (6)	0	3 (10)	7 (25)	2 (2)	3 (5)	4 (36)	5 (23)	<.001
**Posterosuperior (IV) *n* (%)**	5 (9)	3 (9)	0	4 (13)	9 (32)	3 (2)	1 (2)	3 (27)	2 (9)	<.001
**FFM index in neutral position**	0.13 (0.38–1)*	0 (0–0.35)	0 (0–0.63)	0 (0–0.89)*	0 (0–0.35)	0.02 (0–1)*	0 (0–0.95)*	0 (0–0)	0 (0–0)	.04

Continuous variables are presented as median with range in parenthesis; categorical variables are presented as absolute numbers with percentage in parenthesis; Kruskal–Wallis test was used to compare the groups; *:significantly different when comparing with the normal morphology group using the Mann–Whitney *U* test or chi-square test.

**Figure 7:**
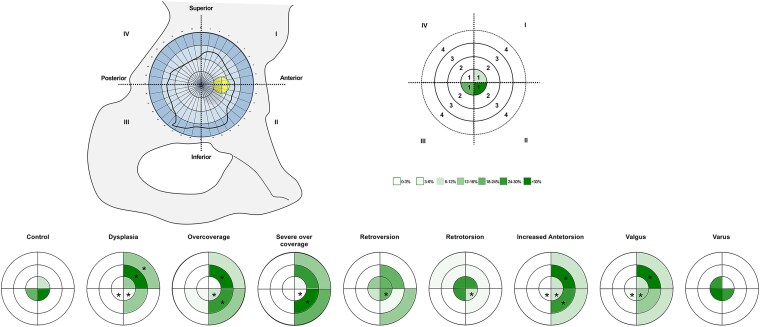
The position of the fovea is shown according to the study groups. *: Significantly different compared with the normal group.

**Figure 8:**
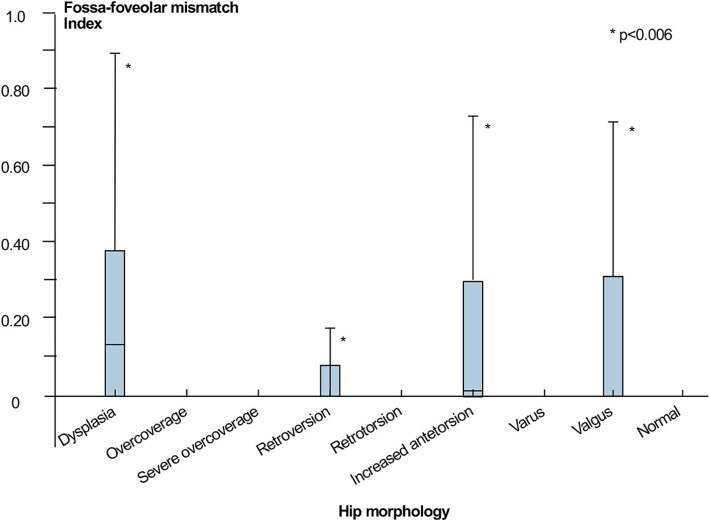
The boxplot demonstrates the FFM indices for all groups. *: Significantly different compared with the normal group.

(2) In the normal morphology group, 100% of the fovea was located within the central radial zone. Within that radial zone, 59% were in the anteroinferior quadrant, 23% were in the posteroinferior quadrant, and 9% were in each of the antero- and posterosuperior quadrants. Each pathomorphologic group exhibited foveal location patterns that were distinct from the normal morphology except the varus subgroup (Table 4; Fig. 7). The varus subgroup demonstrated 100% of the fovea within the central radial zone with 27% in the anteroinferior quadrant, 36% in the posteroinferior quadrant, 9% in the anterosuperior quadrant, and 27% in the posterosuperior quadrant (*P* > .05).

(3) In the normal morphology group, we observed a median FFM index of 0.0 (0.0–0.0). The median FFM index was 0.13 (0.38–1; *P* < .001 compared with normal) for dysplastic hips and 0.02 (0–1; *P* < .001 compared with normal) for hips with increased femoral torsion (Table 4; Fig. 8).

## Discussion

### Proportion of the fovea on the femoral head according to hip morphology

We did not observe significant differences in relative fovea size across the various pathomorphologies, apart from hips presenting retrotorsion where the fovea appeared larger compared with the normal morphology group. This finding suggests that the relative size of the fovea on the femoral head is largely independent of patient-specific hip pathomorphology.

Other authors measured a mean relative fovea size between 17% and 29% [3, 6, 14] using CT scans, AP pelvic radiographs of asymptomatic patients or isolated dry cadaver hips. These values are greater than our findings of 7% in our normal morphology group (Table 5).

**Table 5:** Review table on the fovea capitis femoris dimensions and position from the available literature.

**Author**	**Year**	**Number *n***	**Patient population**	**Age**	**% Male**	**Modality**	**Fovea dimension**	**Fovea position**
Ceynowa [14]	2018	107	Asymptomatic patients	43 y (range 18–60)	50	CT scan	Mean fovea diameter in semicoronal plane: **10.83 mm (SD 2.32).** Mean fovea diameter in transverse plane: **12.94 mm (SD 2.61).** Relative size (Acar’s index): semicoronal plane: **23.05%** (SD 4.69), transverse plane: **27.93%** (SD 5.20) of the femoral head diameter.	Semicoronal plane: The fovea always located **inferiorly to the femoral neck axis in the lower half of the femoral head**. Transverse plane: **75% of the fovea’s diameter posterior to the femoral neck axis.**
Acar [3]	2017	1 200	Patients age between 20 and 80 years, no previous hip surgery, no metabolic bone disease history, no history of hip dysplasia, and no hip-associated pathologies	Group 1 patients 20–39 y *n* = 200; Group 2 patients 40–59 y *n* = 200; and Group 3 patients 60–80 y *n* = 200	50	AP pelvic radiograph, Acars index	FCI (ratio of the fovea size to the femoral head size): mean female general FCI (all ages): **25% ± 4% to 29% ± 5%.** Mean male general FCI (all ages): **27% ± 5% to 29% ± 4%.** Overall FCI (all groups): **26% ± 4% to 29% ± 4%.**	n.a.
Beltran [12]	2012	82	Patients with age 18–43 y with hip pain: dysplastic group, borderline dysplastic group, non-dysplastic group	29 y (range 20–43)	28	MRI on coronal plane with delta angle	n.a.	**Fovea alta in patients with DDH**. Delta angle: DDH: 3.4° ± 12.5° Borderline DDH: 15.7° ± 13.3° Non-DDH: 21.7° ± 12.9°
Perumal [15]	2019	229 (105 European and 124 Thai cadavers)	Cadavers	Overall 70.2 y European: 79.7 y; Thai: 67.6 y	58	Dissection, direct visualization	Mean transverse diameter: **13.9 ± 4.3 mm**; mean longitudinal diameter: **18.1 ± 4.6 mm**	n.a.
Perumal [6]	2017	125	Isolated dry cadaver hips	n.a.	n.a.	Dissection, direct visualization	Mean transverse diameter: **12.8 mm**; mean longitudinal diameter: **18.3 mm**	Fovea consistently located **posteroinferior to the true centre of the femoral head** in 123 out of 125 bones
Sampatchalit [7]	2009	11	Fresh-frozen cadavers with unknown clinical history	76 (range 60–95)	73	MRA	Mean transverse diameter: **26.1 ± 6.5 mm**; mean longitudinal diameter: **33.9 ± 6.7 mm**	n.a.

DDH: developmental dysplasia of the hip; y: years; FCI: fovea capitis index; MRI: magnetic resonance imaging; MRA: magnetic resonance arthrography

A previous study found a larger fovea capitis in degenerated ligaments [7]. However, we did not account for the integrity/degeneration of the LT in our study; therefore, we are unable to determine whether patients with retrotorsion exhibit a higher prevalence of LT lesions.

### Fovea position on the femoral head

We observed that all hips in the normal morphology group were in the central radial zone, located exactly in the middle of the fossa. This central positioning aligns with the expected anatomical distribution in hips without morphologic abnormalities. In contrast, the pathomorphologic groups demonstrated more excentric and diverse fovea positions. Perumal *et al.* reported a slightly posteroinferior location of the fovea capitis femoris in dry isolated adult cadavers (with no information on hip pain) by photographing the femoral head from a mediolateral perspective and using a reference scale [6]. However, their study does not clearly specify the exact viewpoint or methodology used to determine the foveal position, which may limit the reproducibility of their findings. In contrast, our study provides a more detailed evaluation of foveal positioning. Within the normal morphology group, we found that only 23% of foveas were in a posteroinferior position, whereas the majority (59%) were located anteroinferiorly.

The more superior position of the fovea observed in our cohort of dysplastic patients aligns with previously reported findings in the literature. Notably, Nötzli *et al.* [15] reported an abnormally superior location of the fovea in AP pelvic radiographs in patients with hip dysplasia, which they called ‘fovea alta’. Another study utilizing magnetic resonance imaging confirmed the high prevalence of fovea alta in dysplastic patients (Table 5) [4]. However, to the best of our knowledge, the current study is the first to demonstrate a more anterior location of the fovea capitis femoris in dysplastic patients. This novel finding suggests not only a superior displacement but also a potential shift along the sagittal plane. These results add a new dimension to our understanding of foveal pathomorphology in DDH and warrant further investigation into its clinical relevance.

In their CT-based study of asymptomatic patients, Ceynowa *et al.* [14] standardized femoral positioning using the femoral neck axis, aligning the femoral head and neck in semicoronal and transverse planes. While effective, this method does not account for rotational variability caused by femoral torsion. We used the femoral condyles as a reference to standardize rotational orientation. This approach provides a fixed anatomical baseline, reducing the influence of femoral torsion and ensuring more consistent and reproducible measurements of the fovea’s position. While Ceynowa *et al.* [14] reported a posteroinferior location of the fovea capitis femoris, we found it to be predominantly anteroinferior in the normal morphology group. The discrepancies in foveal position in the AP plane are likely because of the different methodologies used.

### Fossa-foveolar mismatch in neutral position

In all groups except for dysplastic hips, the median FFM index was 0, indicating that the entire foveal surface area was located within the acetabular fossa, with no overlap onto the lunate surface. Dysplastic hips had the most excentrically located fovea, with the highest FFM indices. The predominantly anterosuperior peripheral position of the fovea contributes to the elevated FFM indices. It increases the likelihood of contact with the lunate cartilage, not only in the neutral position but also throughout physiological range of motion. This could potentially lead to an LT impingement, in which the ligament is compressed between the fovea capitis and the lunate surface of the acetabulum when it extends beyond the confines of the acetabular fossa. This phenomenon may result in degenerative changes of the structure itself, as well as the acetabular fossa and fovea capitis (Fig. 1). Further research is needed to better understand the clinical implications of dynamic FFM, particularly in individuals with dysplasia.

### Limitations

The present study has limitations. First, our groups did not consist of equal numbers of male and female hips. This may have affected our values regarding the proportions of the femoral head encompassed by the fovea. However, previous studies found no significant differences between males and females with respect to this parameter [3]. Another demographic limitation of our study is the age difference between groups, with the normal morphology group having a significantly higher mean age than the other groups (mean 56 years old *versus* 28 years old, *P* < .001). While one study reported increased fovea size with age, we could not reproduce this finding in our normal morphology group. Further research is needed to clarify the effect of age on foveal size.

Second, to establish a standardized reference, we defined an arbitrary ‘neutral position’ of the hip, by aligning the femoral condyles within the frontal plane, and by placing a femoral head in zero degrees of rotation, ab/adduction and flexion/extension. While this alignment may not necessarily reflect the natural resting position of the hip for every individual, it facilitated consistent and comparable measurements across all samples. We were able to account for femoral torsion, whereas other studies that used the proximal femur as a standardized reference point that did not account for this morphological feature [14].

Nevertheless, we were not able to correct for the functional femoral version, which is the summation of the anatomic femoral version and the functional femoral rotation. The latter is a measurement of how much the femur is rotated around its long axis relative to a functional (typically standing) position. According to literature, this functional femoral rotation is highly variable, ranging from –46.0° (internal rotation) to +37.0° (external rotation) [16]. As we used a standardized ‘artificial’ joint position with the femoral condyles horizontal, we cannot prove whether an LT impingement actually occurs during normal patient activity. Further studies accounting for the functional femoral version in stance and during motion are needed to confirm the pathomechanism in the different hip morphologies. Third, we simplified the shape of the fovea and used circles in our 3D models for all hips. This could have affected the FFM index. Indeed, Perumal *et al.* [6] showed that the fovea could be either circular, oval, or triangular in shape, with the majority being oval (66%).

Fourth, although each hip was independently allocated to a specific study group based on well-defined criteria for acetabular and femoral morphology, the inclusion of bilateral hips from the same patient may have introduced a lack of independence between observations. However, by evaluating each hip separately and using objective criteria for classification, we aimed to minimize this effect.

## Conclusion

This study provides key insights into foveal morphology in normal and pathologic hip morphologies. The surface proportion of the fovea on the femoral head was largely independent of hip pathomorphology, except in hips with deficient femoral torsion that had increased fovea sizes. Foveal position varied significantly, with normal morphology displaying a centralized position and dysplastic hips exhibiting a distinct excentric anterosuperior location.

This altered position in dysplastic hips leads to elevated FFM indices, potentially increasing the risk of contact with the lunate cartilage during motion. Our findings pave the way for new research for the implications of the different foveolar morphologies on the FFM during range of motion and potential lesions.

## Data Availability

The data underlying this article will be shared upon reasonable request to the corresponding author.

## Notes

*Level of evidence:* Non-randomized cohort study; Level of evidence III.
